# Protein structure aids predicting functional perturbation of missense variants in *SCN5A* and *KCNQ1*

**DOI:** 10.1016/j.csbj.2019.01.008

**Published:** 2019-02-01

**Authors:** Brett M. Kroncke, Jeffrey Mendenhall, Derek K. Smith, Charles R. Sanders, John A. Capra, Alfred L. George, Jeffrey D. Blume, Jens Meiler, Dan M. Roden

**Affiliations:** a Department of Medicine, Vanderbilt University Medical Center, Nashville, TN 37232, USA; b Department of Chemistry, Vanderbilt University, Nashville, TN 37232, USA; c Center for Structural Biology, Vanderbilt University, Nashville, TN 37232, USA; d Department of Biostatistics, Vanderbilt University, Nashville, TN 37240, USA; e Department of Biochemistry, Vanderbilt University, Nashville, TN, 37232, USA; f Department of Biological Sciences, Vanderbilt University, Nashville, TN 37235, USA; g Department of Biomedical Informatics, Vanderbilt University Medical Center, Nashville, TN 37235, USA; h Department of Pharmacology, Northwestern University Feinberg School of Medicine, Chicago, IL 60611, USA; i Department of Pharmacology, Vanderbilt University Medical Center, Nashville, TN, 37232, USA

**Keywords:** *SCN5A*, *KCNQ1*, Function prediction, Protein structure, And protein function

## Abstract

Rare variants in the cardiac potassium channel K_V_7.1 (*KCNQ1*) and sodium channel Na_V_1.5 (*SCN5A*) are implicated in genetic disorders of heart rhythm, including congenital long QT and Brugada syndromes (LQTS, BrS), but also occur in reference populations. We previously reported two sets of Na_V_1.5 (*n* = 356) and K_V_7.1 (*n* = 144) variants with in vitro characterized channel currents gathered from the literature. Here we investigated the ability to predict commonly reported Na_V_1.5 and K_V_7.1 variant functional perturbations by leveraging diverse features including variant classifiers PROVEAN, PolyPhen-2, and SIFT; evolutionary rate and BLAST position specific scoring matrices (PSSM); and structure-based features including “functional densities” which is a measure of the density of pathogenic variants near the residue of interest. Structure-based functional densities were the most significant features for predicting Na_V_1.5 peak current (adj. R^2^ = 0.27) and K_V_7.1 + KCNE1 half-maximal voltage of activation (adj. R^2^ = 0.29). Additionally, use of structure-based functional density values improves loss-of-function classification of *SCN5A* variants with an ROC-AUC of 0.78 compared with other predictive classifiers (AUC = 0.69; two-sided DeLong test *p* = .01). These results suggest structural data can inform predictions of the effect of uncharacterized *SCN5A* and *KCNQ1* variants to provide a deeper understanding of their burden on carriers.

## Graphical abstract

**Figure:**
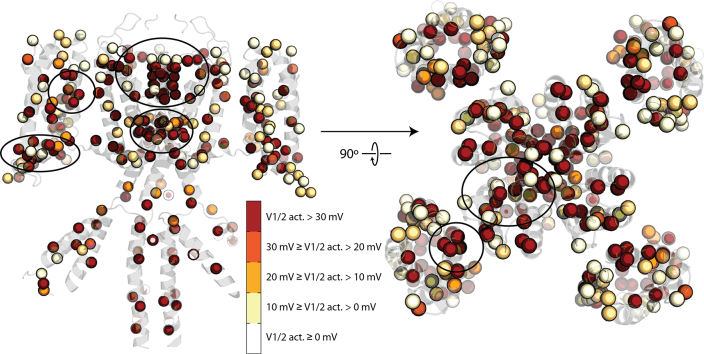

## 1 Introduction

Of an estimated 20,000 nonsynonymous single nucleotide polymorphisms (nsSNPs) in each individual's protein-coding genome, approximately 10 are presently predicted to be clinically actionable [26]. nsSNPs in *KCNQ1* (K_V_7.1 channel protein, which complexes with the protein KCNE1 to generate the slow cardiac potassium repolarization current, I_Ks_) and *SCN5A* (Na_V_1.5 channel protein, which generates the cardiac depolarizing sodium current, I_Na_), are associated with heritable diseases of the heart [4,36,37,38,49] including dilated cardiomyopathy [14,28], cardiac conduction disease [6,29], short QT syndrome [13], sick sinus syndrome [15], types 1 and 3 congenital long QT syndromes (LQTS) [7,18,30,38], and Brugada syndrome (BrS) [5]. However, in aggregate, rare nsSNPs in *SCN5A* and *KCNQ1* also appear at ~2% in the population, being more common than the rare arrhythmia disorders associated with these genes, suggesting only limited roles in disease. Determining the significance and effect size of these nsSNPs will be of increasing importance as more people undergo genome or exome sequencing [3,27].

Models used to predict the effect of these nsSNPs are most commonly trained on the information-poor inputs of binary disease-inducing/benign classification. Binary classification reduces information. Moreover, the disease-inducing vs. benign distinction ignores penetrance and the underlying molecular phenotype—or potentially multiple overlapping molecular phenotypes—that may be most informative for therapy. A striking example involves patients presenting with type 3 long QT syndrome due to a gain-of-function *SCN5A* variant that also impairs trafficking of the encoded channel Na_V_1.5. Therapeutic targeting of this gain-of-function with the antiarrhythmic drug mexiletine can increase cell surface expression of the mutant channel, leading to the unintended consequence of exaggerating the long QT phenotype [34,35,46].

Using literature datasets we have recently curated for both I_Ks_ [25,47] and I_Na_, [22] we test the hypothesis that incorporating variant-specific functional features from *KCNQ1* and *SCN5A* nsSNPs and structure-based features into prediction models will improve our ability to predict if previously uncharacterized nsSNPs will result in altered currents. Secondary structural elements are independent predictors of deleterious variants in *SCN5A* and can improve current prediction models [20], suggesting the potential utility of structure-based approaches. In fact, the highest densities of disease-associated variants across the *entire* spectrum of proteins fall largely in structured, functional segments: the structure/function of these molecules are compromised in the disease state [23,50]. Here, we generated a set of models able to predict I_Na_ and I_Ks_ variant-specific current phenotypes. Identifying the variant-specific functional perturbation will provide an additional tool to geneticists and physicians to determine if variants are likely disease-causing and to more accurately stratify the degree of risk that carriers who present without a phenotype will eventually develop channelopathy-based heart disease.

## 2 Methods

### 2.1 Quantified functional parameters of KCNQ1 and SCN5A chosen for analysis

For I_Na_, we analyzed peak current, steady state V_1/2_ activation and inactivation, late/persistent current, and recovery from inactivation [49]. For I_Ks_, we analyzed peak current, V_1/2_ activation, and activation and deactivation time constants [19]. We selected these functional features because these parameters are most consistently reported in the literature. We only included functional data from K_V_7.1 variants when functional protocols involved homotetrameric mutated K_V_7.1 coexpressed with KCNE1, since this protocol was most commonly reported in the literature. Details about how each dataset was collected is contained in the original papers.([22,25]; C. G. [48]) Briefly, all variants were normalized to WT measurements included in the same publication, i.e. peak current mutant/peak current WT, or V_1/2_ activation (mutant) – V_1/2_ activation (WT), etc.

Most functionally characterized variants in SCN5A were characterized by heterologous expression in human embryonic kidney cells (291 of 356 total), so we used only patch-clamp data derived in human embryonic kidney cells when available. For KCNQ1-KCNE1, most variants were characterized in CHO cells (79 of 165 total). We averaged the individual parameters in cases where multiple articles reported functional characterization of the same variant in the same cell system.

### 2.2 Generating structural models of K_V_7.1 (KCNQ1)

No experimental structure of transmembrane domains of human K_V_7.1 exists, so we generated models using the recently released *Xenopus* structure of a closed pore and open voltage sensor and the human sequence NP_000209.2 with 91% identity [45]. We used comparative modeling within the Rosetta scripts utility in Rosetta 3.8 to build K_V_7.1 [44]. We rebuilt loops on K_V_7.1 monomers, followed by rebuilding the functional homotetramer with symmetry for 1000 models. Most best-scoring structures had reasonable C_α_ RMSDs between 1 and 3. We selected the best scoring model for subsequent analysis. We built models both with, and without, human calmodulin (CaM) bound; however no significant differences were observed in structure-based features, therefore, we selected K_V_7.1 with CaM bound for the analysis presented here.

### 2.3 Generating structural models of Na_V_1.5

We generated two human Na_V_1.5 structural models using the human sequence NP_000326.2 with the American cockroach sodium channel Na_V_PaS structure [41] (45% identity), and electric eel Na_V_1.4 structure [52] (67% identity). Models of Na_V_1.5 were refined with small, unstructured segments rebuilt using established protocols as for K_V_7.1, generating 1000 models. Most best-scoring structures had reasonable C_α_ RMSDs between 2 and 4. We selected the best scoring model for subsequent analysis. We tested the performance of structure-based features using both models, with very similar results. Because models based on the Na_V_PaS structure allow the inclusion of more variants in the analysis, we report here features calculated using those structural models.

### 2.4 Summary of predictive features

Our objective was to predict variant-specific functional perturbations for the cardiac ion channels K_V_7.1 + KCNE1 (I_Ks_) and Na_V_1.5 (I_Na_). We used the variant classifier models PROVEAN [9], PolyPhen-2 [1], and SIFT [24]; sequence alignment-based rate of evolution [32], and mutation rates derived from BLAST position specific scoring matrices (PSSM), and Point Accepted Mutation (PAM) matrix score [39]; and several structure-based features including burial propensities (how often certain residues are in the interior of the protein), neighbor counts (number of neighboring residues), neighbor identities (propensity of neighboring residues to be close in space) and what we term functional density (k-nearest neighbors-inspired metric to estimate functional perturbation). These predictive features are described below and summarized in Table S1. As can be seen in the higher off-diagonal R^2^s, predictive classifiers were modestly degenerate; functional density weight only, i.e. the local enrichment for variants that had been functionally characterized, were more degenerate (described below, Figs. S1 and S2).

### 2.5 Calculating structure-derived features

NeighborCount is derived from the number of nearest neighbors weighted by distance and within 11.4 Å of the residue of interest, a cutoff found to be optimized to predict protein structure [12]. NeighborVector is a variation of neighbor density, scaled by how evenly distributed the nearest neighbor residues are to the residue of interest. Amino acid neighbor count (aaneigh) and amino acid neighbor vector (aaneighvector) are analogous to NeighborCount and NeighborVector, respectively, modified to account for amino acid-specific propensities for a given degree of burial [12,51]. NeighborCount, NeighborVector, aaneigh, aaneighvector predictive features were generated using the BioChemical Library (BCL) and the structures described above (for more detail see [12,51]).

### 2.6 Generating a structure-based functional density predictor

In addition to the structure-based features described above, we leveraged both the structural models and variant-specific functional datasets for I_Ks_ and I_Na_ in estimating the “functional density”. Using an approach akin to k-nearest neighbors, we calculated functional density by averaging functional perturbations of variants near the variant of interest weighted by the inverse of their distance from the variant of interest. This calculated feature therefore depends on how many functionally perturbed variants are near the variant of interest, with regions in three-dimensional space dense with functionally perturbed variants—“hotspots”—yielding a more perturbed prediction. However, all functionally characterized variants contribute to this parameter. We did not use a cutoff to determine whether or not to include a variant in this analysis. Functional density is calculated as follows:

$$\rho_{j,x}=\frac{\sum_{i=0}^{n}∆{\mathrm{function}}_{x,i,\mathrm{mutation}\left(i\right)}∙\frac{1}{1+e^{\left(\frac{d_{i,j}}{2}\right)}}}{\sum_{i=0}^{n}\frac{1}{1+e^{\left(\frac{d_{i,j}}{2}\right)}}}$$

where ρ_j_ is functional density of the j^th^ residue and x^th^ functional parameter, Δfunction_x,i_ is the change in functional parameter x for the i^th^ variant, and d_i,j_ is the distance between the center of mass of residues i and j. i does include residue j, but only if the identity of the amino-acid mutation is changed, i.e. mutation(i) ≠ mutation(j). A graphical representation is shown in Fig. S3. The distribution of neighboring residues is similar between K_V_7.1 and Na_V_1.5, with a first shell of contacting residues at ~6 Å and a second shell at ~11 Å (Fig. S4). Additionally, we calculated the functional density weights alone (same equation as above, but with *∆function* = 1) to test whether signal derived from functional densities could be attributed to protein region bias in the variants that have been functionally characterized.

### 2.7 Variant-specific I_Na_ and I_Ks_ functional perturbation predictive models

Because the number of features in our dataset was large relative to the number of variants, regularization was used to fit predictive models. We used a fully relaxed LASSO penalty, which has good predictive performance overall [16]. Prediction models were 10-fold cross-validated. After feature selection, the relaxed generalized linear model was bootstrapped (1000 times) to obtain bootstrapped percentile intervals for quantities of interest. We report the adjusted coefficient of determination, adj. R^2^, with 95% confidence intervals as a measure of overall prediction of the relaxed LASSO model. We focused on models where LASSO shrinkage yielded at least one significant predictive feature and the lower bound of the naïve 95% confidence interval for the adj. R^2^ was >0.10. Relatively few models were able to meet these minimum criteria. Note that since the functional density features were calculated from the data, we additionally subjected the fully relaxed LASSO to higher-level 10-fold cross validation procedure which included a functional density construction step. This accounts for any variability or overfitting that might result from using data-determined functional covariates.

### 2.8 Loss-of-function classification of I_Na_ and I_Ks_ with and without structure-based features

We further classified loss-of-function variants by degree of functional perturbation, for I_Na_ defined as <50% peak current [22] and for I_Ks_ < 50% peak current or > 10 mV positive shift in V_1/2_ activation [25], to estimate the impact of functional densities on this task. We used commonly available variant sequence-based classifiers PolyPhen2, PROVEAN, BLAST-PSSM, and rate of evolution individually, all combined, and all combined with peak current functional density in a logistic regression model. We generated 95% confidence intervals on AUCs from the candidate models using bootstrap with 2000 replicates and used a two-sided DeLong test to evaluate ROC difference significance.

## 3 Results

### 3.1 Ion channel missense variants have diverse effects on current

**Fig. 1:**
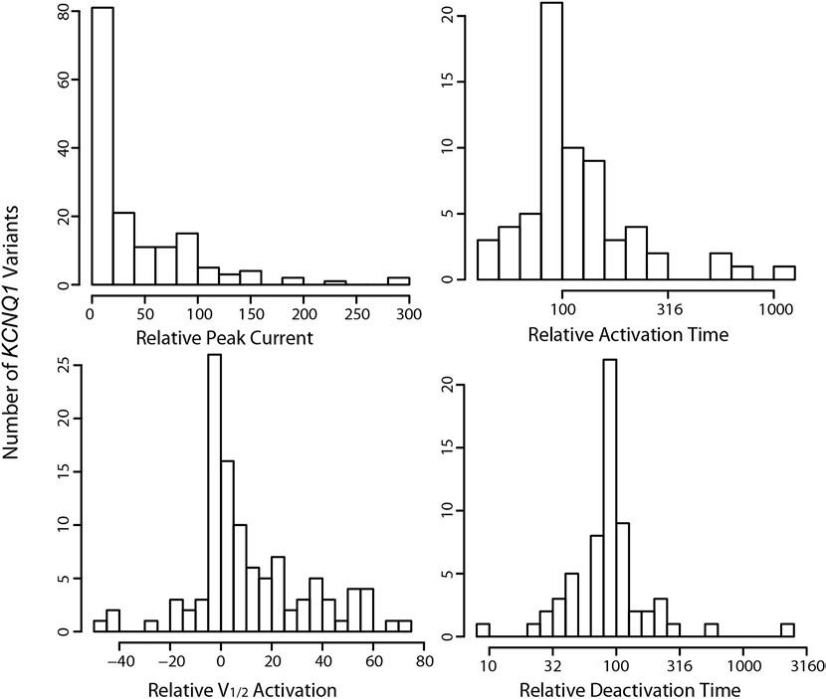
Histogram distributions of all functional parameters for K_V_7.1 + KCNE1 (I_Ks_) analyzed in this paper. All values are referenced to WT which is either 100% or 0 mV.

**Fig. 2:**
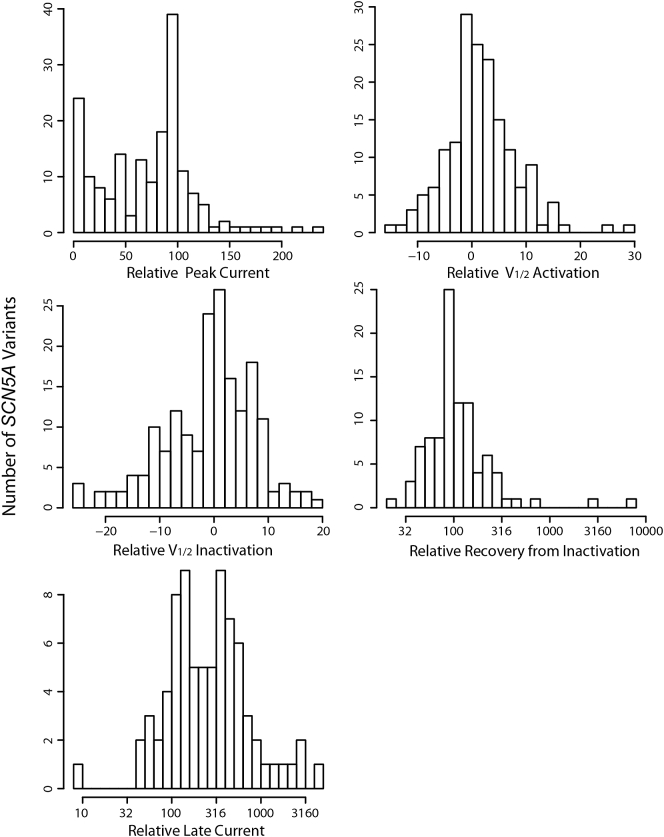
Histogram distributions of all functional parameters for Na_V_1.5 (I_Na_) analyzed in this paper. All values are referenced to WT which is either 100% or 0 mV.

Histograms of all functional parameters analyzed are shown in Figs. 1 and 2 and Table 1. For homotetrameric K_V_7.1 variants, the distribution of I_Ks_ current maxima is skewed towards 0% current compared to WT function, likely a reflection of literature bias. The distribution of I_Na_ variant current maxima is bimodal with centers at 0% (complete LOF) and 100% (WT). I_Ks_ V_1/2_ activation is also skewed towards more positive values, whereas I_Na_ V_1/2_ activation is more evenly distributed about 0 mV. I_Na_ late current is skewed towards higher values. Time constants for I_Ks_ activation and inactivation and I_Na_ recovery from inactivation are clustered around WT with very wide ranges, populated with few points at extremely long characteristic times.

**Table 1:** Summary statistics of functional parameters.

Na_V_1.5	# of variants	Median [1st Q, 3rd Q]	WT
Peak Current	162	82 [36, 100] (%WT)	100%
Late Current	61	253 [122, 474] (%WT)	100%
V_1/2_ Activation	163	0.00 [−1.63, 3.09] (mV)	0 mV
V_1/2_ Inactivation	141	0.00 [−4.00, 3.44] (mV)	0 mV
Inactivation Recovery	85	98 [76, 138] (%WT)	100%

K_V_7.1	# of variants	Median [1^st^ Q, 3^rd^ Q]	WT

I_Ks_peak	142	17 [0, 59] (%WT)	100%
V_1/2_ Act	93	6.40 [0.00, 23.80] (mV)	0 mV
tau_act	58	106 [94, 150] (%WT)	100%
tau_deact	57	87 [70, 115] (%WT)	100%

### 3.2 Models can significantly predict I_Na_ and I_Ks_ peak current but rely on different predictive features

**Fig. 3:**
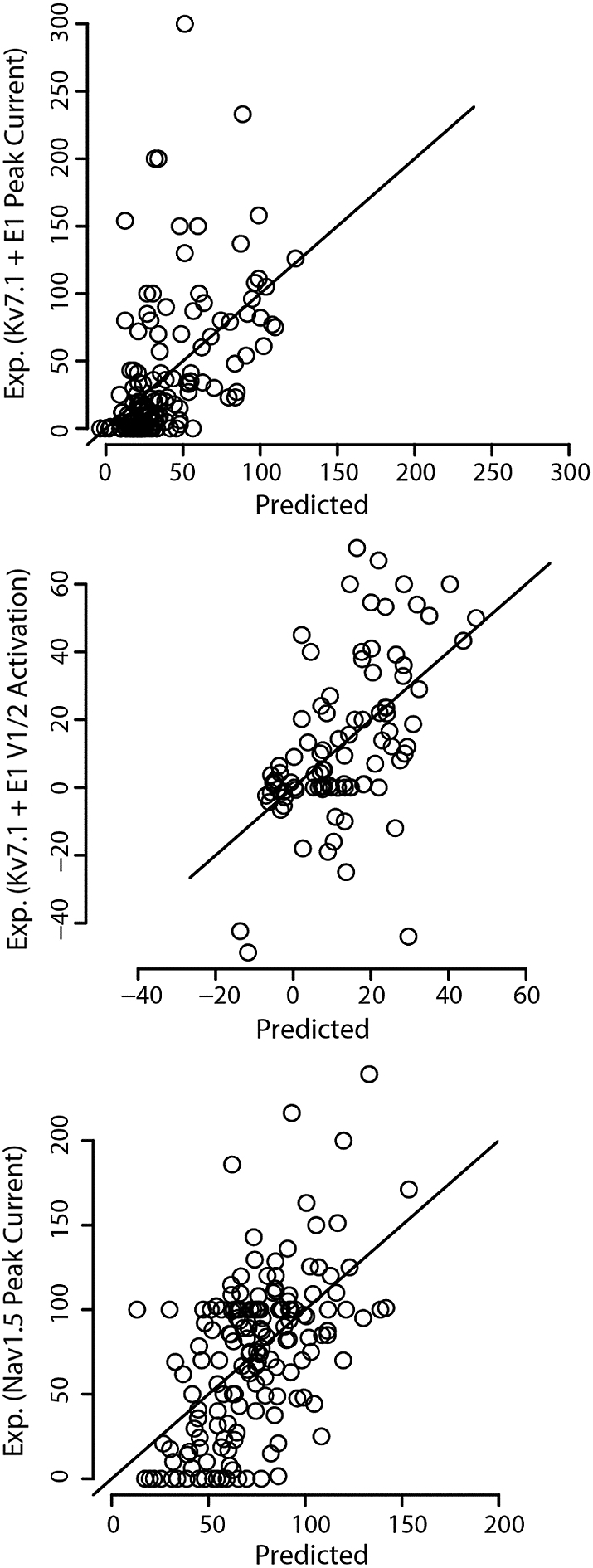
Experimental vs. predicted functional parameters for the subset of functional features with significant predictive models (Table 2). Plot of experimental I_ks_ peak current, I_ks_ V_1/2_ activation, and I_Na_ Na_V_1.5 peak current vs. predictions from a linear regression. The resulting models explain 0.24, 0.29, and 0.27 of the variance in I_ks_ peak current, I_ks_ V1/2 activation, and I_Na_ peak current, respectively.

Using a linear model, we could predict peak current, a proxy for overall channel function, for both I_Ks_ and I_Na_ (lower bound 95% CI adj. R^2^ of 0.14 and 0.18 respectively; Table 2 and Fig. 3). Interestingly, sequence-based predictors, especially BLAST-PSSM, had the most significant association with I_Ks_ peak current (Table S2, Fig. S5) but were not as integral to predicting I_Na_ peak current (Table S3, Fig. S6). Conversely, functional density for peak current provided most of the signal for I_Na_ but did not contribute meaningfully to I_Ks_ peak current prediction. This suggests a spatial dependence of peak current for I_Na_ not recapitulated by other published predictive models, contrary to I_Ks_. This difference may be due in part to the comparatively large fraction of reported *SCN5A* variants that do not perturb peak current yet are still associated with cardiac diseases compared to *KCNQ1*, such as LQT3 variants with increased late current but no change in peak current; BLAST-PSSM is sensitive to evolutionary fitness of residue changes which may be more homogeneously dependent on peak current for *KCNQ1* and more heterogeneous for *SCN5A*. Alternatively, the spatial distribution of I_Ks_ peak current may be more heterogeneous than for I_Na_. The functional density weight, a measure of the number of functionally characterized variants proximal to a residue of interest, was selected out of the I_Ks_ peak current model, but not for I_Na_ suggesting a modest sampling bias in regions of Na_V_1.5 sensitive to peak current perturbation.

**Table 2:** Summary statistics of predictive model.

Functional parameter	Adj. R2† [95% CI†; CV‡]
I_Ks_ Peak Current	0.24 [0.14–0.46; 0.24]
I_Ks_ V_1/2_ Activation	0.29 [0.12–0.48; 0.23]
Na_V_1.5 Peak Current	0.27 [0.18–0.45; 0.23]
Na_V_1.5 V_1/2_ Inact.	0.16 [0.08–0.34; 0.05]

† CI (confidence interval)

‡ CV (10 fold cross-validation)

### 3.3 Models can predict steady-state I_Ks_ V_1/2_ activation but not I_Na_ V_1/2_ activation or inactivation

We were able to significantly model I_Ks_ V_1/2_ activation. However, no models could reliably predict I_Na_ V_1/2_ activation or inactivation. The I_Ks_ V_1/2_ activation variance explained is relatively high, 0.29 with a 95% confidence interval lower bound of 0.12 (Table 2). The functional density feature had a significant *p*-value, suggesting a three-dimensional localization of regions that influence V_1/2_ activation (Table S2, Fig. S7).

### 3.4 Most I_Na_ and I_Ks_ functional parameters cannot be reliably predicted

Most I_Ks_ and I_Na_ functional parameters assessed could not be predicted with stable fully relaxed LASSO-regularized linear models and a lower bound of the 95% confidence interval in adj. R^2^ >0.10. In many cases for these functional parameters, at least one of the 10 folds in the cross validation resulted in only an intercept, i.e. β coefficients for all inputted features shrunk to 0. For some functional parameters, such as time constants for I_Ks_ activation and inactivation and I_Na_ late current and recovery from inactivation times, lower numbers of characterized variants and relatively low dispersion of values (Table 1, Figs. 1 and 2) mean the data themselves are limiting prediction. Alternatively, or in addition, our chosen feature set may contain little information relevant to the prediction of these values, likely the case for I_Na_ V_1/2_ activation and inactivation, which may be under sampled for the functional density analysis.

### 3.5 Structural features improve I_Na_ but not I_Ks_ loss-of-function classification

For comparison with published variant classifiers predicting binary functional perturbation of these two channels [22,25], we calculated receiver operating characteristic curves for models trained using only published models as features and models trained additionally with structure-based features. We generated binary classifications of loss-of-function *SCN5A* and *KCNQ1* variants using criteria described above in the methods section. We calculated the ability of several variant classifiers to correctly classify LOF variants. The resulting areas under the curve (AUCs) from logistic models trained to predict *KCNQ1* LOF were as follows (AUC; [95% CI]): PolyPhen-2 (0.81; [0.74–0.92]), rate of evolution (0.77; [0.67–0.87]), BLAST-PSSM (0.84; [0.76–0.92]), PROVEAN (0.83; [0.75–91]), all published predictive models (0.86; [0.78–0.94]), all published predictive models with functional density for peak current (0.87; [0.79–0.94]). Most variant classifiers performed reasonably well and the addition of structural information did not meaningfully improve classification for this task. However, the resulting AUCs from logistic models trained to predict *SCN5A* LOF were as follows: PolyPhen-2 (0.60; [0.51–0.68]), rate of evolution (0.51; [0.42–0.60]), BLAST-PSSM (0.61; [0.52–0.69]), PROVEAN (0.66; [0.57–0.75]), SIFT (0.53; [0.48–0.58]), all published variant classifiers (0.69; [0.60–0.77]), all published variant classifiers with functional density for peak current (0.78; [0.70–0.85]). This improvement in classification ability for LOF variants in *SCN5A* when adding functional density for peak current (0.69 without vs. 0.78 with, *p* = .01) suggests structure-based features contribute information not contained in other predictive features (Fig. S8) an observation gaining appreciation elsewhere [[42], [43]].

## 4 Discussion

### 4.1 A limited number of I_Ks_ and I_Na_ functional parameters can be predicted reliably

Most I_Ks_ and I_Na_ functional parameters analyzed could not be predicted reliably: I_Ks_ time constants of activation and inactivation; and I_Na_ V_1/2_ activation/inactivation, recovery from inactivation, and late current. However, three important functional parameters could be predicted: I_Ks_ peak current and V_1/2_ activation and I_Na_ peak current. In two of these models, I_Ks_ V_1/2_ activation and I_Na_ peak current, the functional density features have the greatest predictive value, indicating three-dimensional enrichment of regions of the proteins that influence these functional parameters (Table S2 and S3, Figs. S6 and S7).

### 4.2 Functional density suggests regions in three-dimensional space are enriched for influence on I_Ks_ V_1/2_ activation and I_Na_ peak current

**Fig. 4:**
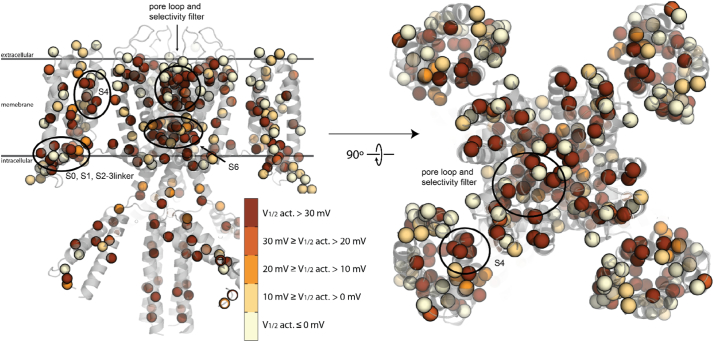
Structural model of K_V_7.1 with colored spheres at C_α_ positions where variants have V_1/2_ activation data available. Colors indicate the degree of perturbation from WT K_V_7.1, with the darker color displaying variants with more positive shifts in V_1/2_ activation. Selection criteria are displayed in the inset. Several regions of apparent enrichment are highlighted by circles. The tetrameric structure gives the appearance of a greater number of functionally characterized variants.

**Fig. 5:**
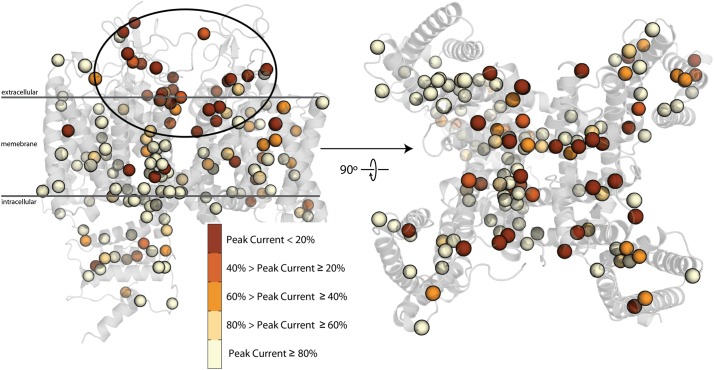
Structural model of Na_V_1.5 with colored spheres at C_α_ positions where variants have peak current available. Colors indicate the degree of perturbation from WT Na_V_1.5, with the darker color displaying variants with less peak current. Selection criteria are displayed in the inset. A single extracellular region shows apparent enrichment and is circled. Even though there are a greater number of variants functionally characterized for Na_V_1.5, K_V_7.1 appears to have a greater number due to its homotetrameric structure.

“Functional densities” are measure of how dense pathogenic variants are near the residue of interest, i.e. are they near “hotspots” that influence a particular function. Given the influence of the functional density calculation in predicting I_Ks_ V_1/2_ activation and I_Na_ peak current, there is likely a spatial influence over both of these parameters. As can be seen in Figs. 4 and 5, there are regions (black circles) where variants that have a large influence on I_Ks_ V_1/2_ activation and I_Na_ peak current are localized. Not surprisingly, the greatest perturbations in I_Ks_ V_1/2_ activation are in the regions of the channel known to be functionally critical: the selectivity filter, voltage-sensing helix in the voltage sensing domain, and in the constriction point in the middle of the pore, as we have seen previously. [25] The S6 helix in K_V_7.1 influences activation in part through its intrinsic flexibility, a necessary property for activation. [40] S0 helix has been found to provide stabilization to the voltage sensing domain. [17] S4 helix is canonically responsible for voltage-dependent activation [8,11,31]. Interestingly, the variants most disruptive to I_Na_ peak current are located in the extracellular region of the channel, mostly near the selectivity filter. The pore region of voltage-gated sodium channels is canonically responsible for Na^+^ conduction [2] and is also enriched BrS1 variants, an Na_V_1.5 loss-of-function disorder [21,22]. These data suggest the utility in leveraging combined structural and previously determined functional perturbation datasets to predict functional disruption of previously uncharacterized channel variants.

### 4.3 Challenging regions to predict

To identify potential commonalities among the most challenging variants to predict, we identified the five least congruent predictions, at extremes both greater and less than experiment, for I_Ks_ peak current, I_Ks_ V_1/2_ activation, and I_Na_ peak current (Fig. S8–10). All variants, with one exception, occur in the transmembrane region and on structured segments, not flexible loops or linkers. Some commonalities for challenges in predicting I_Ks_ peak current and V_1/2_ activation prediction are the extracellular half of the voltage sensing domain, especially S3 and S4 helices, and the interface between the pore loop helix and helices S5 and S6. The S3 and S4 helices of the voltage sensing domain undergo large conformational changes in response to voltage [8,11,31] which are not captured by the static structure we used in this analysis. However, the distribution of predictions both greater than and less than experiment within these two segments suggests changes in function in these regions are heterogeneous possibly due to individual residues in these regions having special roles in voltage-gated activation. Interestingly, several of the challenging I_Ks_ peak current variants are located on the S0 helix in K_V_7.1. We previously observed an anomalous sensitivity to expression level in the S0 helix and suggest the protein is stabilized by intramolecular interactions between the S0 helix and the rest of the voltage sensing domain. [17] Challenging variants for I_Na_ peak current are more evenly distributed though the protein molecule (Fig. S10).

### 4.4 Classification of loss-of-function KCNQ1 and SCN5A variants

Classification of variants inherently reduces the richness of available data, in our case the continuous functional perturbation induced by variants in *SCN5A* and *KCNQ1.* However, to assess how well structure-based features contribute to predicting variant loss-of-function classification, we built logistic models trained on variants classified as loss-of-function or not loss-of-function. For I_Na_, structure-based features improve the AUC (Fig. S11); for I_Ks_ there is no significant improvement. This is consistent with our previous *KCNQ1* work suggesting sequence and evolutionary-based features, BLAST-PSSM and residue rate of evolution, yield a competent classification model and suggests alternative features will be needed to further improve prediction of *KCNQ1* variants [25]. For *SCN5A,* structure-based features improve the classification of loss-of-function variants from an AUC of 0.69 to 0.78 (*p* = .01).

### 4.5 Recent interest in predicting functional perturbation

Recently. Clerx et al. attempted to predict classification of functionally compromised I_Na_ for many of the functional parameters we report here [10]. The authors report modest classification ability for I_Na_ late current and V_1/2_ activation/inactivation with better performance predicting complete loss of function. We too find limited ability to predict most functional perturbations; however, we found significant and *quantitative* correlations between predicted and experimental I_Na_ peak current and challenge the use of functional classification in favor of quantitative perturbation prediction. Interestingly, the authors also noted difficulty in predicting late current which we recapitulate here suggesting this feature is a more challenging target to predict. Furthermore, here we put forward a feature based on knowledge of the three-dimensional structure, functional density, and demonstrate its utility in predicting variant phenotype.

### 4.6 Application to variant annotation

The field is still evolving on how to include in silico predictions and experimental functional data quantitatively [33]. We suggest the model presented here could be useful in a pipeline whose first-pass filter aims to detect pathogenic variants. Our previous publication suggested the degree to which a loss-of-function variant produces non-negligible penetrance was an I_Na_ peak current 50% or less than that of WT. We suggest this implies the need to have a variance explained of experimental data from our predictions >50% such that the probability a variant predicted to be WT actually has <50% peak current is very low. Predicting around 0.2 of the variance in relevant I_Ks_ and I_Na_ functional parameters we show here is significant; however, further improvement is needed before the predictive models will be useful in classifying variants for clinical use.

### 4.7 Limitations

The dataset used was limited by those variants available in the literature, which are biased towards functionally perturbed variants. We chose to analyze I_Ks_ generated with homozygous K_V_7.1 variants (co-expressed with KCNE1) because this configuration is reported most consistently in the literature. In a majority of cases, K_V_7.1 variants are heterozygous in individuals. Furthermore, we have begun to investigate the influence of variant-specific functional perturbation on clinical presentation [22], but the exact relationship is complicated (notably including β-adrenergic regulation for I_Ks_) and warrants further investigation. Another limitation is that the structural models are imperfect estimates of the functional state they represent and are also only representative of a single functional state in channels known to have at least two functional states. Models reflecting greater conformational diversity may be another source for improved features.

### 4.8 Conclusions

We have derived predictive features from three-dimensional structures of Na_V_1.5 and K_V_7.1 and have demonstrated these features improve our ability to predict variant-induced functional perturbations in each channel. These predictive features are based on recognizing that residue positions for pathogenic variants are likely to be clustered in three-dimensional space in proximity to other pathogenic residues. Based on this recognition, we can account for approximately 0.2 of the variance in I_Ks_ peak current, I_Ks_ V_1/2_ activation, and I_Na_ peak current. For I_Ks_ V_1/2_ activation and I_Na_ peak current, structure-based features contribute meaningfully to the predictive model and in a way not recapitulated by commonly used sequence, evolutionary features, or genetic variant classifiers methods. For predicting variant-induced loss-of-function, structure-based features contribute meaningfully to I_Na_ but not I_Ks_.

## Funding

This work was supported by the National Institutes of Health K99HL135442 to B.M.K.; R35GM127087 to J.A.C.; HL122010 to A.L.G., C.R.S., and J.M.; and P50GM115305 to D.M.R.

## Appendix A Supplementary data

Supplementary material 1 Image 1

Supplementary material 2 Image 2

Supplementary material 3 Image 3
