# Supplementary material for: Protein structure aids predicting functional perturbation of missense variants in SCN5A and KCNQ1
Source: Comput Struct Biotechnol J. 2019 Feb 1;17:206–14. doi: 10.1016/j.csbj.2019.01.008 (PMC6383132; doi:10.1016/j.csbj.2019.01.008)
Supplement: Supplementary file 1 — Supplementary material 1 [file mmc1.docx]

Supplemental Materials: Protein structure aids predicting functional perturbation of missense variants in *SCN5A* and *KCNQ1*

Brett M. Kroncke,^1^* Jeffrey Mendenhall,^2,3^ Derek K. Smith,^4^ Charles R. Sanders,^3,5^ John A. Capra,^6,7^ Alfred L. George, Jr.,^8^ Jeffrey D. Blume,^4^ Jens Meiler,^2,3,9^ and Dan M. Roden, MD^1,7,9^

# **Tables**

| **Table S1. Features used to predict functional perturbation** | | |
| --- | --- | --- |
| **Feature** | **Description** | |
| NeighborCount | Structure-based feature involving the number of neighbors within 11.4 Å of the residue of interest |  |
| NeighborVector | Structure-based feature involving the density and directionality of nearest neighbor residues to the residue of interest. |  |
| aaneigh | Structure-based feature similar to NeighborCount, but modified to account for amino acid-specific propensities for a given degree of burial |  |
| aaneighvector | Structure-based feature similar to NeighborVector, but modified to account for amino acid-specific propensities for a given degree of burial |  |
| PROVEAN | Protein Variation Effect Analyzer (PROVEAN) uses pairwise sequence alignment scores to predict functional effects of genetic variants.^1^ |  |
| SIFT | Sorting Intolerant From Tolerant (SIFT): perturbation predictions based on sequence homology and changes in amino-acid chemistry.^2^ |  |
| PolyPhen-2 | Polymorphism Phenotyping v2 (PolyPhen-2) uses naïve Bayes classification based on a number of predictive features including sequence, phylogenetic and structural information.^3^ |  |
| PAM score | Point Accepted Mutation (PAM30 matrix) scores amino-acid substitutions based on likelihood of 30 residues per 100 randomly changing over time.^4^ |  |
| rate of evolution | Estimate the rate of evolution per residue using a multiple sequence alignment of homologues and the Rate4site method as previously reported.^5, 6^ |  |
| BLAST-PSSM | BLAST position specific scoring matrices derived from sequence alignments from the NCBI non-redundant sequence database^7^ with PSI-BLAST.^8^ |  |
| Functional Density | average functional perturbations of variants near the variant of interest weighted by the inverse of the distance away, as defined above. |  |

| **Table S2. Chunk test p-values for predictive features in linear models of Kv7.1 + KCNE1 (I_ks_)** | | | | |
| --- | --- | --- | --- | --- |
| **Feature** | **I_ks_ peak** | **I_ks_ V_1/2_ Act** | |  |
| Density V_1/2_ Activation | - | | 1.78x10^-6^ |  |
| Density Peak Current | 0.064 | | - |  |
| BLAST-PSSM | 0.335 | | - |  |
| PolyPhen-2 | 0.118 | | 0.049 |  |
| PROVEAN | 0.785 | | - |  |

| **Table S3. Chunk test p-values for features in a linear model of Nav1.5 (I_Na_)** | | |
| --- | --- | --- |
| **Feature** | **I_Na_ Peak** |  |
| Density Peak Current | 7.19x10^-07^ |  |
| Density Peak Current (weights) | 0.016 |  |
| PAM score | 0.025 |  |
| BLAST-PSSM | 0.934 |  |
| PROVEAN | 0.887 |  |
| SIFT | 0.263 |  |
| PolyPhen-2 | 0.085 |  |
| AANeigh | 0.684 |  |
| AANeighVector | 0.596 |  |
| NeighborVector | 0.081 |  |
| Rate of Evolution | 0.456 |  |

# **Figure Legends**

Figure S1. Heatmap of Pearson R^2^ for experimental and predictive features for Kv7.1. “Calculated” functional parameters were calculated using the functional density algorithm (see methods section). Evolution rate BLAST-PSSM, PROVEAN, and PolyPhen-2 are sequence alignment based-features from online tools. PAM is an amino acid-based feature. All other features are structure based and described in the methods section. “Weight only” designates functional density calculation with $\Delta function$ = 1 and therefore reflects the tendency in 3D space for variants to be overrepresented in the literature.

Figure S2. Heatmap of Pearson R^2^ for experimental and predictive features for Nav1.5. “Calculated” functional parameters were calculated using the functional density algorithm (see methods section). Parameters defined as for Figure S1.

Figure S3. Schematic of weighted distance distribution used to calculate functional density.

Figure S4. The density of neighboring residues as a function of distance, normalized to the area of a sphere. There is a peak around 6 Å for both Na_V_1.5 and K_V_7.1 at the first shell surrounding packed residues, subsequent shells are observed at ~11 Å and ~15 Å, but are much less in phase.

Figure S5. Most influential predictive features to predict I_ks_ peak current. Individual features influencing peak current prediction, peak current functional density and BLAST-PSSM, are plotted vs experiment with a locally weighted average (solid line) and 95% CI on the weighted average (dashed line).

Figure S6. Most influential predictive features to predict Nav1.5 peak current, peak current functional density and PAM score. Solid and dashed lines defined as in Figure S5.

Figure S7. Most influential predictive features to predict I_ks_ V_1/2_ activation, V_1/2_ activation functional density and PolyPhen-2. Solid and dashed lines defined as in Figure S5.

Figure S8. The five least congruent predictions for I_Ks_ peak current, at extremes greater than experiment, were (in order of most distant from experiment) T104I, V110I, I227L, V310I, and E160K (red); predictions less than experiment were V141M, S209P, R259H, R109L, and P197L (blue).

Figure S9. Predictions for I_Ks_ V1/2 greater than experiment were H258R, V307L, L236P, F279I, and S209F; predictions less than experiment were G269S, L236R, K557E, V310I, and R231H.

Figure S10. Predictions for I_Na_ peak current greater than experiment were I1660V, S401L, T187I, Q1832E, and M1766L; predictions less than experiment were N1774D, R225P, F1616Y, S401N, and F1473C.

Figure S11. Receiver operating characteristic curves. Logistic regression models trained with and without structure-based features, determined to be significant by the LASSO model on predicting loss-of-function classification of variants in *SCN5A* and *KCNQ1* as detailed in the discussion. All models include all *in silico* predictive models and excludes structure-based features.

# **Figures**

Figure S1


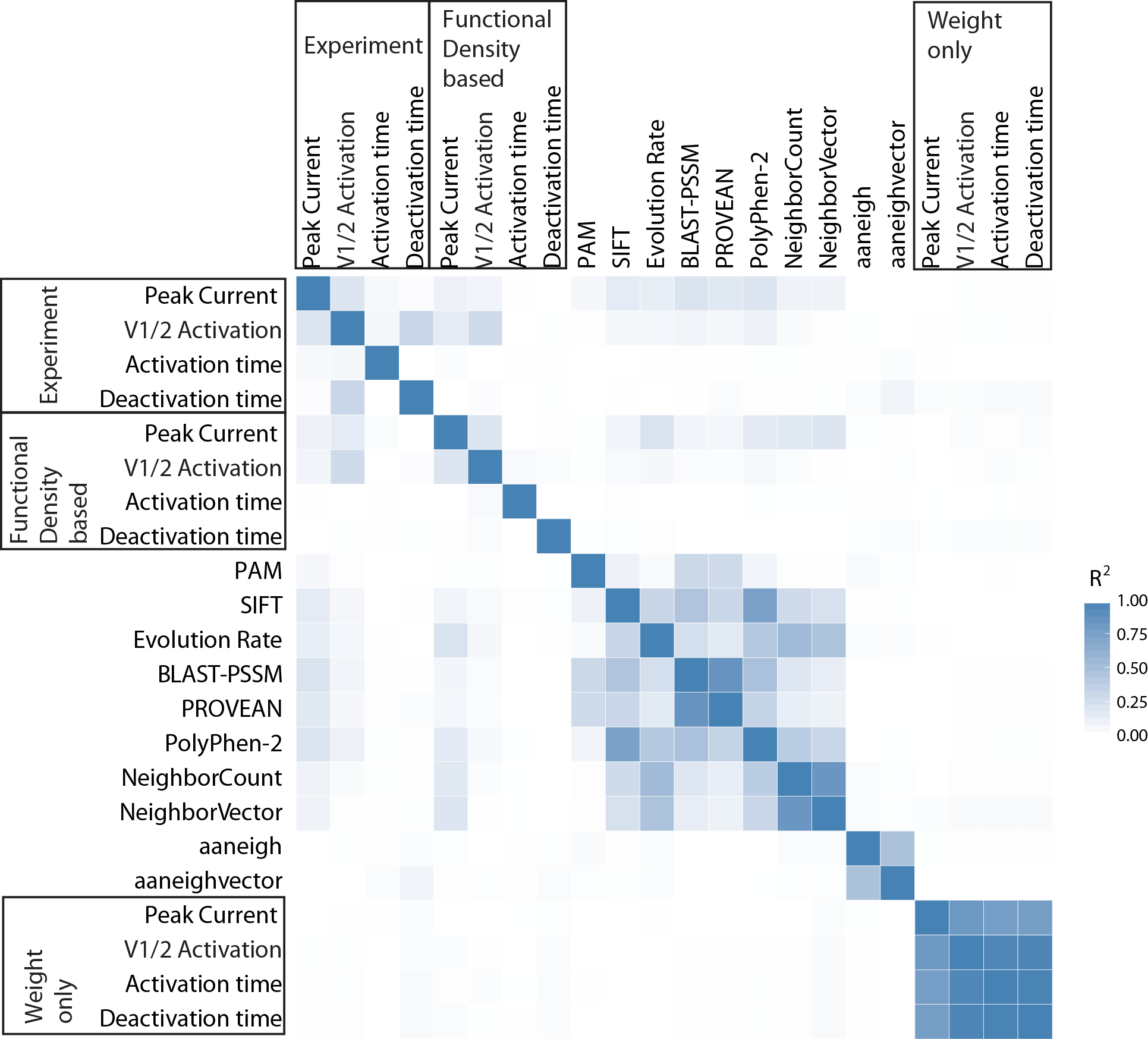


Figure S2


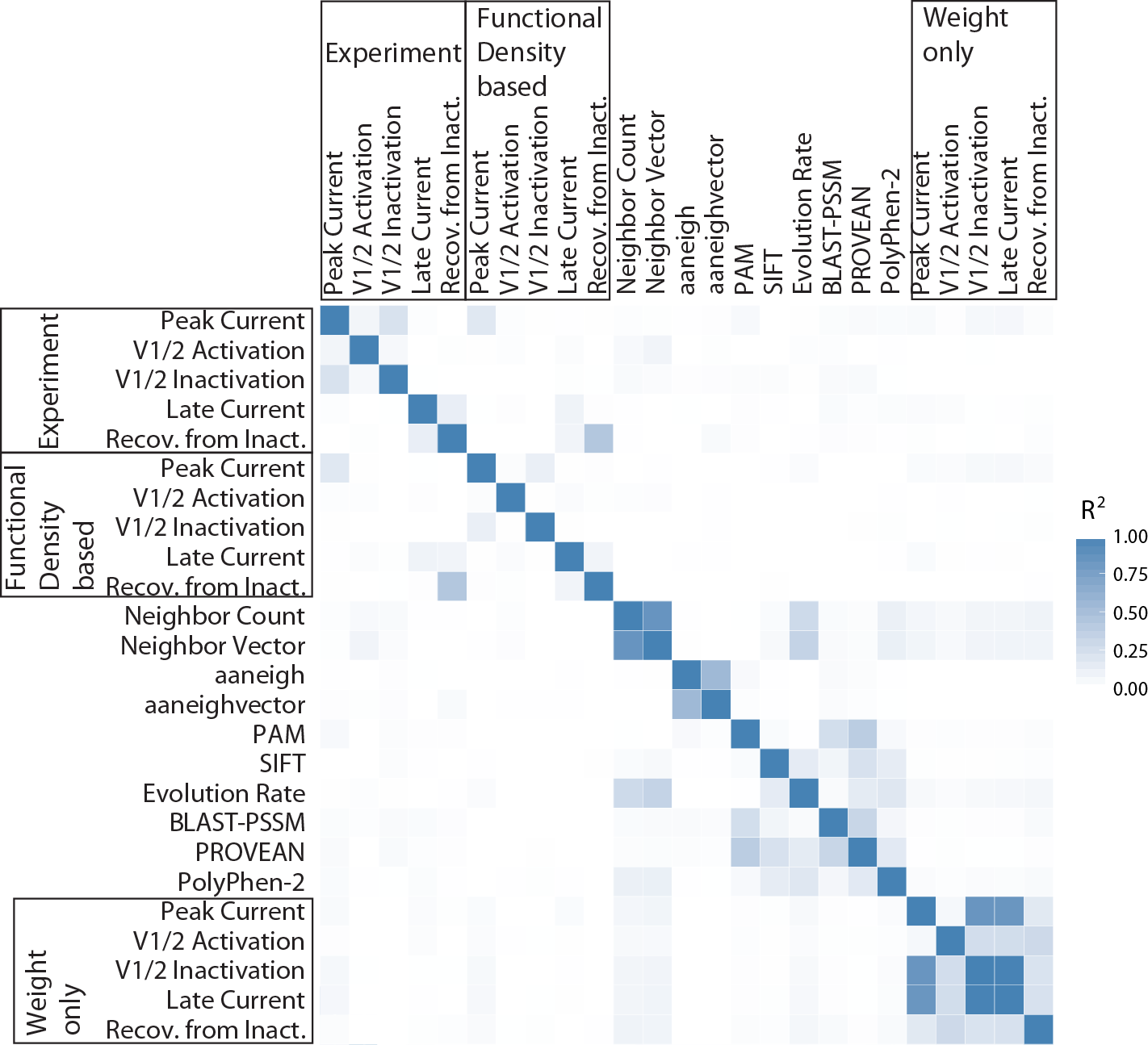


Figure S3


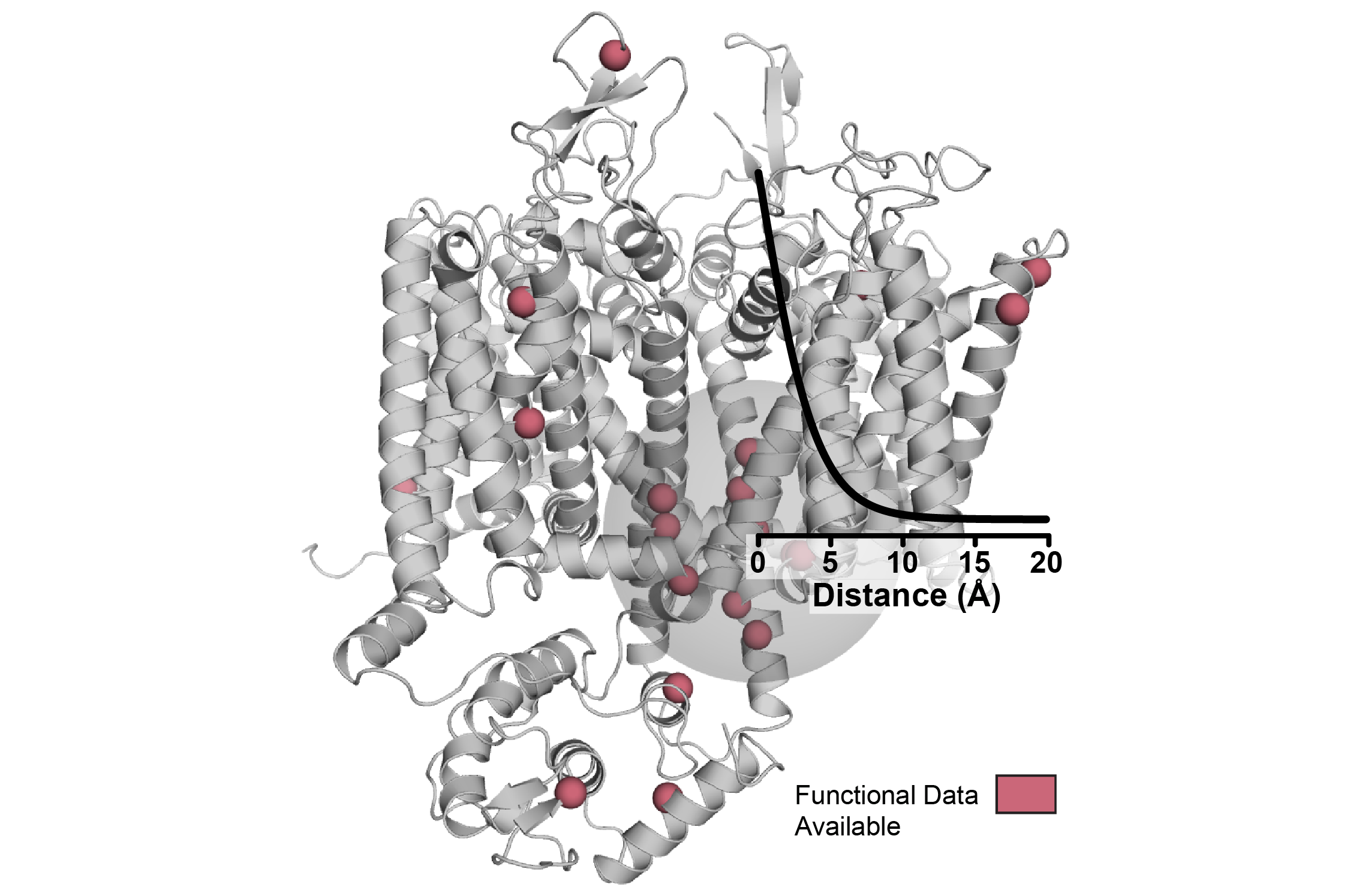


Figure S4


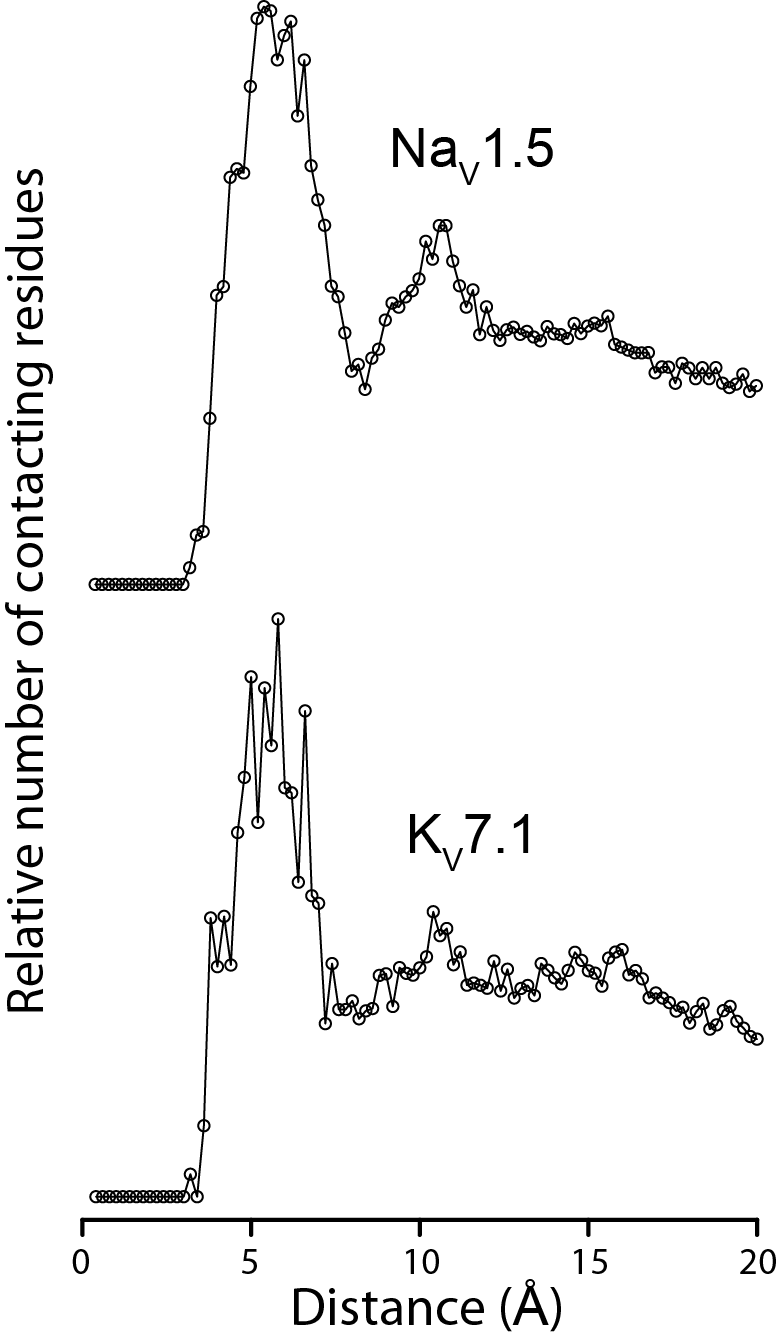


Figure S5


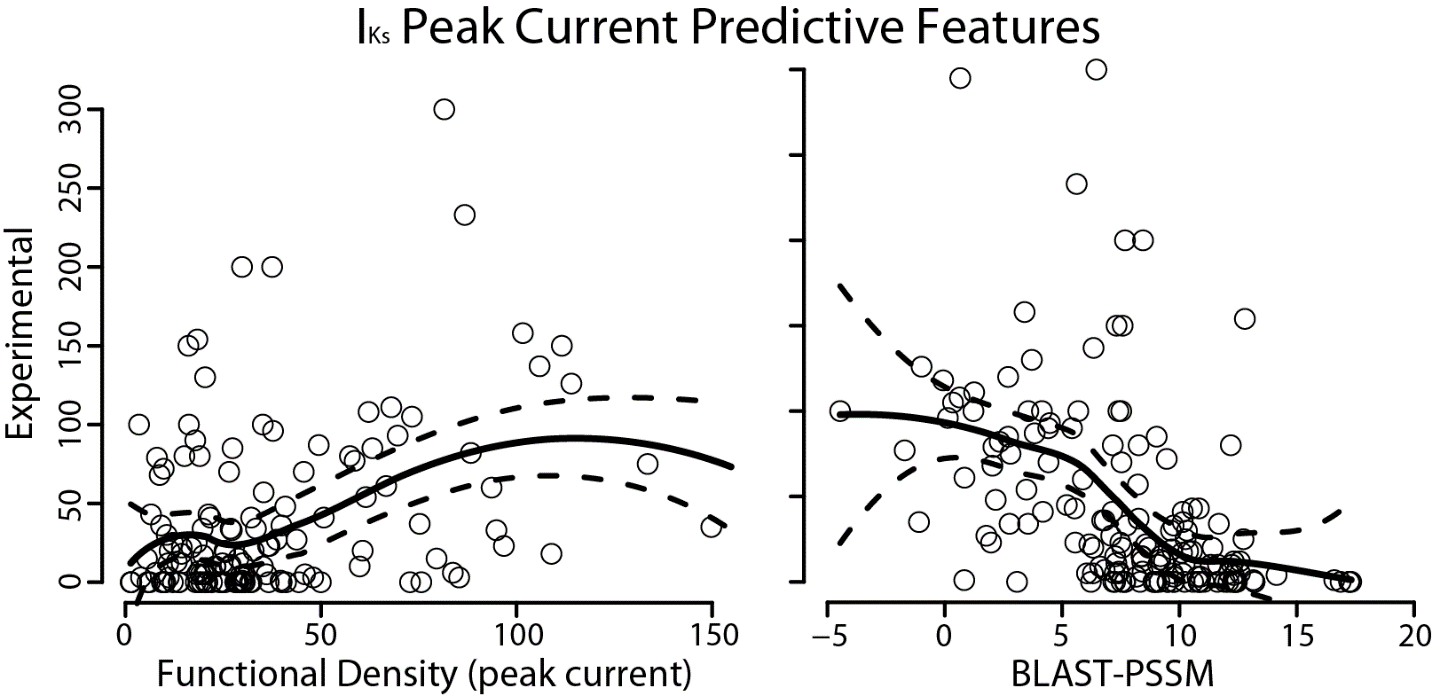


Figure S6
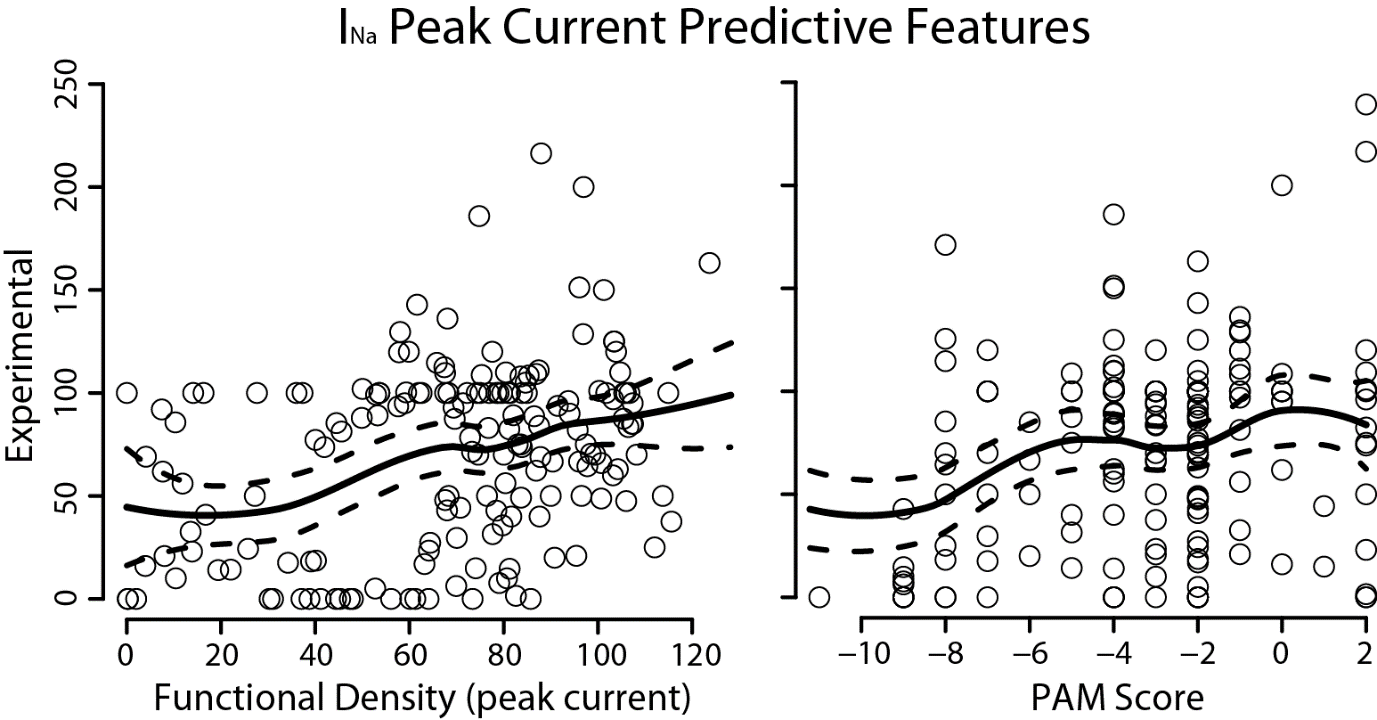


Figure S7


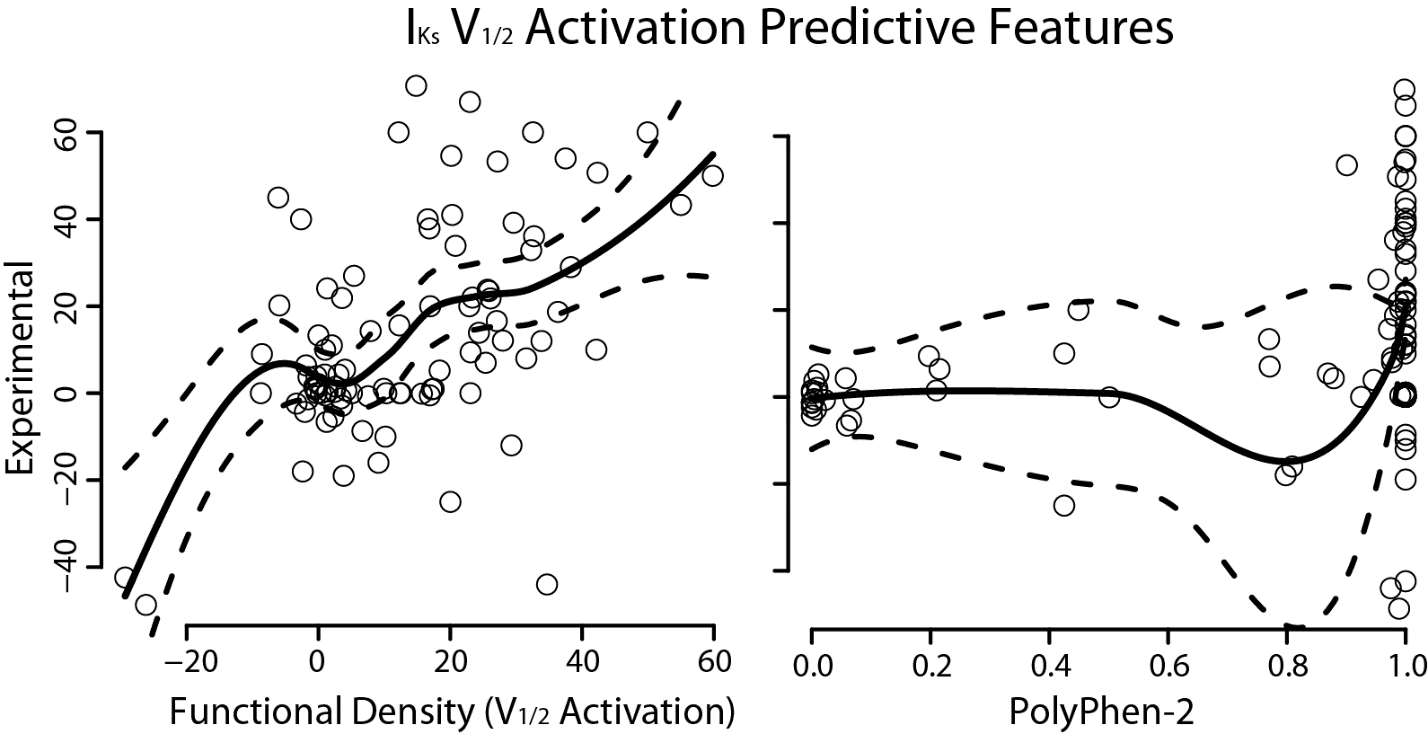


Figure S8


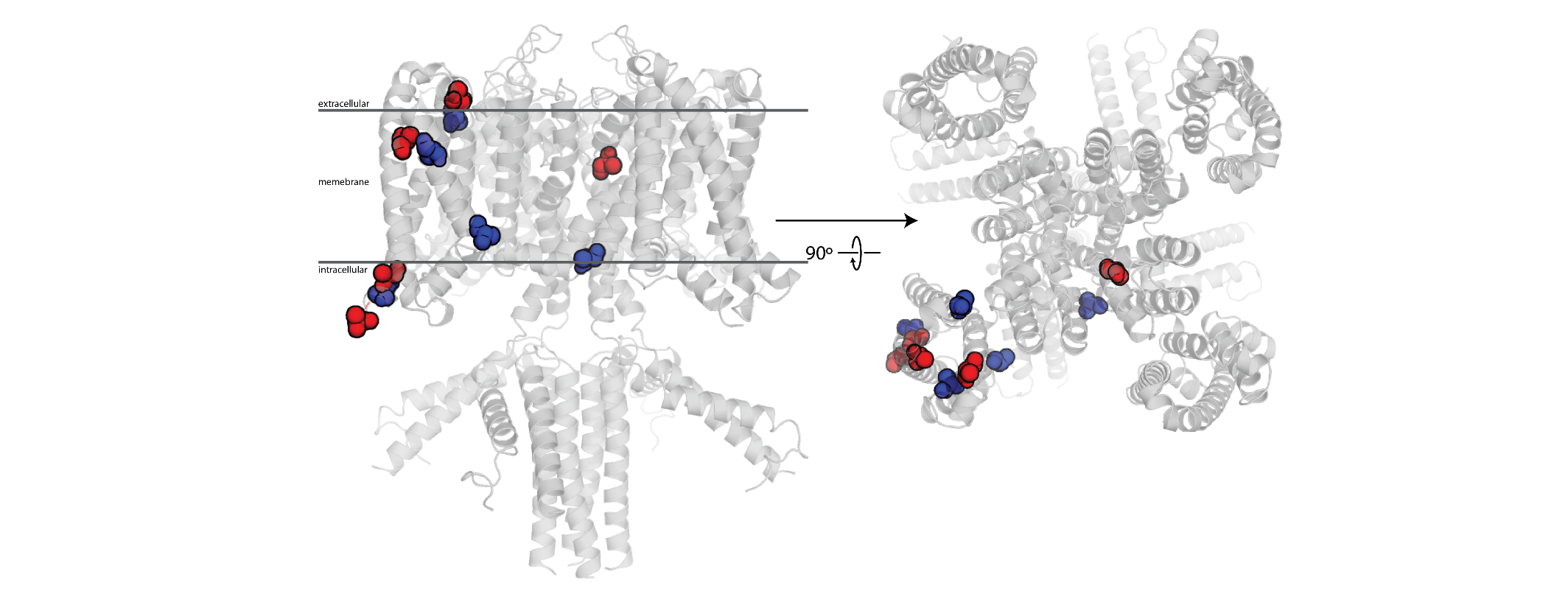


Figure S9


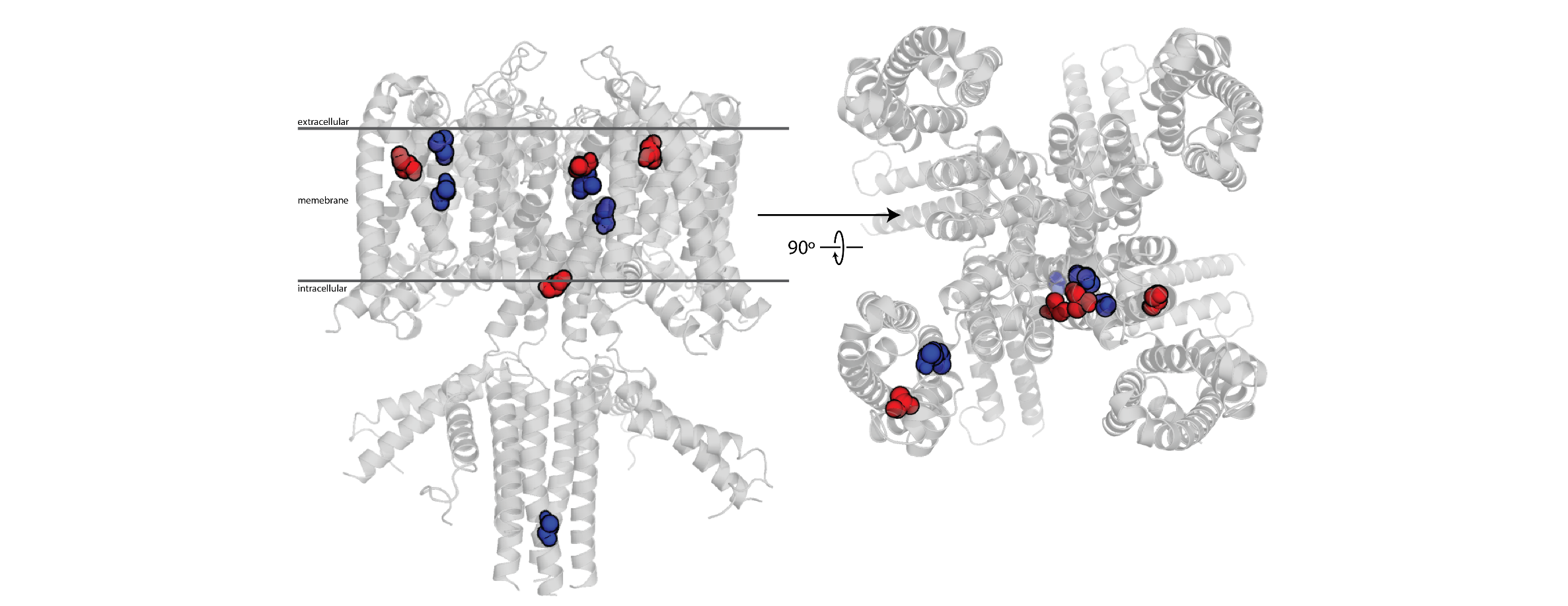


Figure S10


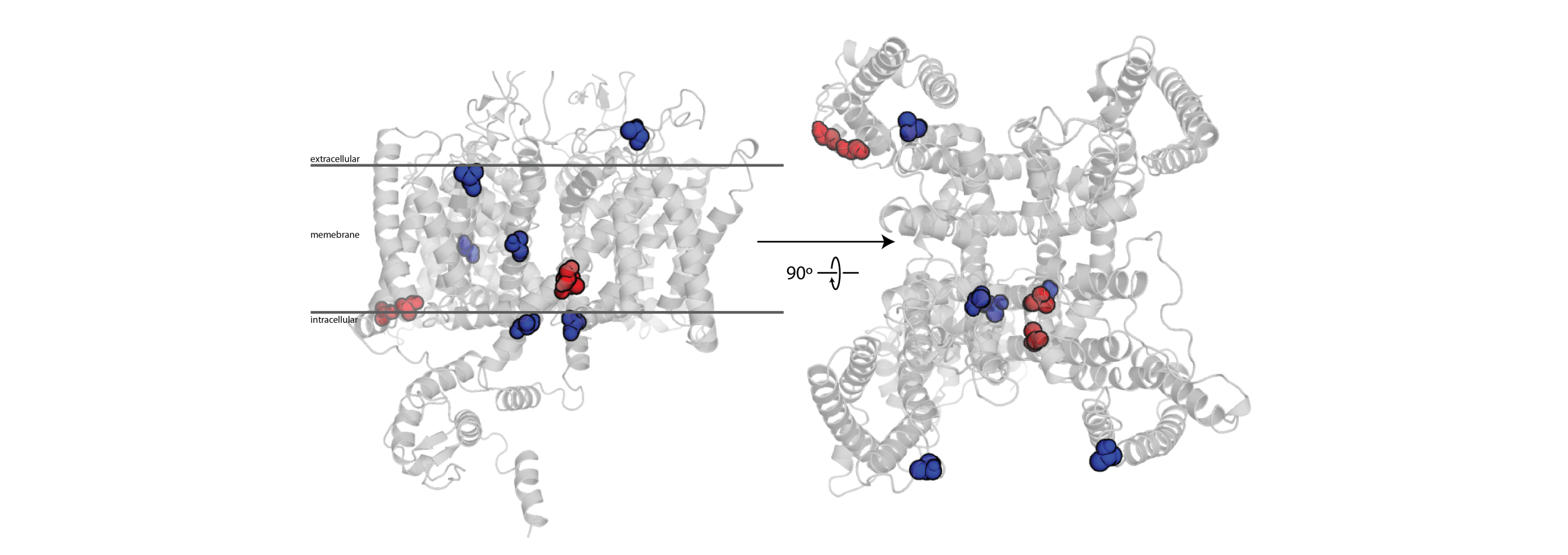


Figure S11


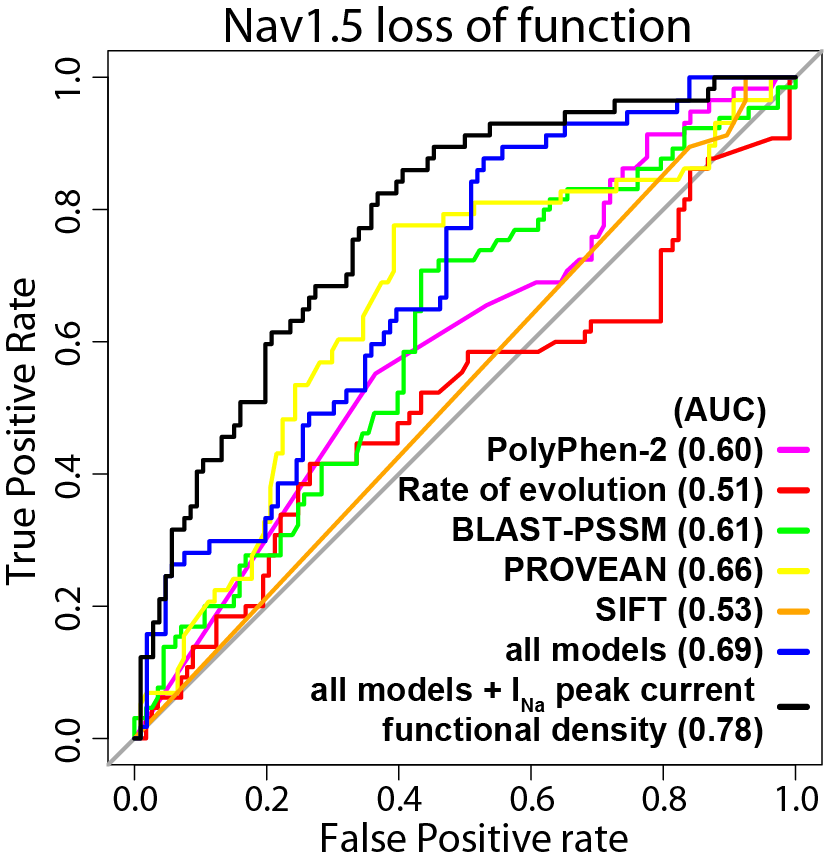


# **Methods**

Functional density is calculated as follows where ρ_j_ is the functional density of the j^th^ residue, Δfunction_x,i_ is the change in functional parameter x for the ith residue, and d_i,j_ is the distance between the center of mass of residues i and j. i does include residue j, but only if the identity of the amino-acid mutation is changed, i.e. mutation(i) ≠ mutation(j). A graphical representation is shown in Figure S3.

$$\rho_{j}=\sum_{i=0}^{n} {\Delta function}_{x,i,mutation\left( i \right)}\cdot\frac{1}{1+e^{(\frac{d_{i,j}}{2})}}$$

# **References**

1. Choi Y, Sims GE, Murphy S, Miller JR ,Chan AP. Predicting the functional effect of amino acid substitutions and indels. *PloS one*. 2012;7:e46688.

2. Kumar P, Henikoff S ,Ng PC. Predicting the effects of coding non-synonymous variants on protein function using the SIFT algorithm. *Nat Protoc*. 2009;4:1073-81.

3. Adzhubei IA, Schmidt S, Peshkin L, Ramensky VE, Gerasimova A, Bork P, et al. A method and server for predicting damaging missense mutations. *Nat Methods*. 2010;7:248-9.

4. Schwarz R ,Dayhoff M. Matrices for detecting distant relationships. In: M. Dayhoff, ed. *Atlas of protein sequences*: National Biomedical Research Foundation; 1979: 353-358.

5. Li B, Mendenhall JL, Kroncke BM, Taylor KC, Huang H, Smith DK, et al. Predicting the Functional Impact of KCNQ1 Variants of Unknown Significance. *Circ Cardiovasc Genet*. 2017;10.

6. Pupko T, Bell RE, Mayrose I, Glaser F ,Ben-Tal N. Rate4Site: an algorithmic tool for the identification of functional regions in proteins by surface mapping of evolutionary determinants within their homologues. *Bioinformatics*. 2002;18 Suppl 1:S71-7.

7. Pruitt KD, Tatusova T ,Maglott DR. NCBI reference sequences (RefSeq): a curated non-redundant sequence database of genomes, transcripts and proteins. *Nucleic Acids Res*. 2007;35:D61-5.

8. Altschul SF, Madden TL, Schaffer AA, Zhang J, Zhang Z, Miller W, et al. Gapped BLAST and PSI-BLAST: a new generation of protein database search programs. *Nucleic Acids Res*. 1997;25:3389-402.
